# Self-rated health in individuals with and without disease is associated with multiple biomarkers representing multiple biological domains

**DOI:** 10.1038/s41598-021-85668-7

**Published:** 2021-03-17

**Authors:** L. Kananen, L. Enroth, J. Raitanen, J. Jylhävä, A. Bürkle, M. Moreno-Villanueva, J. Bernhardt, O. Toussaint, B. Grubeck-Loebenstein, M. Malavolta, A. Basso, F. Piacenza, S. Collino, E. S. Gonos, E. Sikora, D. Gradinaru, E. H. J. M. Jansen, M. E. T. Dollé, M. Salmon, W. Stuetz, D. Weber, T. Grune, N. Breusing, A. Simm, M. Capri, C. Franceschi, P. E. Slagboom, D. C. S. Talbot, C. Libert, S. Koskinen, H. Bruunsgaard, ÅM. Hansen, R. Lund, M. Hurme, M. Jylhä

**Affiliations:** 1 Faculty of Social Sciences (Health Sciences), Tampere University, Tampere, Finland; 2 Gerontology Research Center (GEREC), Tampere, Finland; 3 Faculty of Medicine and Health Technology (MET), Tampere University, Tampere, Finland; 4 Department of Medical Epidemiology and Biostatistics, Karolinska Institutet, Stockholm, Sweden; 5 Molecular Toxicology Group, University of Konstanz, Konstanz, Germany; 6 BioTeSys GmbH, 73728 Esslingen, Germany; 7 Research Unit On Cellular Biology, University of Namur, Rue de Bruxelles, 61, 5000 Namur, Belgium; 8 Research Institute for Biomedical Aging Research, University of Innsbruck, Rennweg, 10, 6020 Innsbruck, Austria; 9 Advanced Technology Center for Aging Research, Scientific Technological Area, IRCCS INRCA, Ancona, Italy; 10 Nestlé Research, Nestlé Institute of Health Sciences, EPFL Innovation Park, 1015 Lausanne, Switzerland; 11 Institute of Biology, Medicinal Chemistry and Biotechnology, National Hellenic Research Foundation, Athens, Greece; 12 Laboratory of the Molecular Bases of Ageing, Nencki Institute of Experimental Biology, Polish Academy of Sciences, 3 Pasteur Street, 02-093 Warsaw, Poland; 13 Carol Davila University of Medicine and Pharmacy, Bucharest, Romania; 14 National Institute for Public Health and the Environment (RIVM), Centre for Health Protection, P.O. Box 1, 3720 BA Bilthoven, The Netherlands; 15 Straticell, Science Park Crealys, Rue Jean Sonet 10, 5032 Les Isnes, Belgique; 16 Institute of Nutritional Sciences (140), University of Hohenheim, 70593 Stuttgart, Germany; 17 Department of Molecular Toxicology, German Institute of Human Nutrition Potsdam-Rehbruecke (DIfE), Nuthetal, Germany; 18 Department of Nutritional Toxicology, Friedrich Schiller University Jena, Dornburger Str. 24, 07743 Jena, Germany; 19 Institute of Nutritional Medicine (180), University of Hohenheim, 70593 Stuttgart, Germany; 20 Department of Cardiothoracic Surgery, University Hospital Halle, Ernst-Grube Str. 40, 06120 Halle (Saale), Germany; 21 DIMES- Department of Experimental, Diagnostic and Specialty Medicine, ALMA MATER STUDIORUM, CIG-Interdepartmental Center “L.Galvani”, University of Bologna, 40126 Bologna, Italy; 22 Department of Applied Mathematics of the Institute of ITMM, National Research Lobachevsky State University of Nizhny Novgorod, Nizhny Novgorod, Russian Federation; 23 Section of Molecular Epidemiology, Leiden University Medical Centre, Leiden, The Netherlands; 24 Unilever Science and Technology, Beauty and Personal Care, Sharnbrook, UK; 25 Center for Inflammation Research, VIB, Ghent, Belgium; 26 Department of Biomedical Molecular Biology, Ghent University, Ghent, Belgium; 27 National Institute for Health and Welfare, Helsinki, Finland; 28 Department of Clinical Immunology, Rigshospitalet, University Hospital of Copenhagen, Copenhagen, Denmark; 29 Department of Public Health, University of Copenhagen, Copenhagen, Denmark; 30 National Research Centre for the Working Environment, Copenhagen, Denmark; 31 Center for Healthy Aging, University of Copenhagen, Copenhagen, Denmark

**Keywords:** Predictive markers, Health care, Quality of life, Somatosensory system

## Abstract

Self-rated health (SRH) is one of the most frequently used indicators in health and social research. Its robust association with mortality in very different populations implies that it is a comprehensive measure of health status and may even reflect the condition of the human organism beyond clinical diagnoses. Yet the biological basis of SRH is poorly understood. We used data from three independent European population samples (N approx. 15,000) to investigate the associations of SRH with 150 biomolecules in blood or urine (biomarkers). Altogether 57 biomarkers representing different organ systems were associated with SRH. In almost half of the cases the association was independent of disease and physical functioning. Biomarkers weakened but did not remove the association between SRH and mortality. We propose three potential pathways through which biomarkers may be incorporated into an individual’s subjective health assessment, including (1) their role in clinical diseases; (2) their association with health-related lifestyles; and (3) their potential to stimulate physical sensations through interoceptive mechanisms. Our findings indicate that SRH has a solid biological basis and it is a valid but non-specific indicator of the biological condition of the human organism.

## Introduction

Self-rated health (SRH) is one of the most frequently used measures in epidemiological, clinical and social research. It is known to predict mortality, future functional status and outcome of treatment in populations that vary by age, gender, social class, health status, country and culture^1,2^. In many studies SRH remains a significant predictor of mortality after adjustment for several other health indicators^2^. However, the wider the array of objectively measured health variables, the weaker is the independent predictive power of SRH^3,4^. Therefore it is plausible that the association between SRH and mortality is not causal but is due to the ability of the self-assessment to more exhaustively capture the realm of `health` and the objective bodily condition than most other health indicators^2,5^.

Jylhä (2009) has suggested that when asked to evaluate their general health status, respondents will take into account any individual relevant information that they think describes their “health”. This information is then considered in the context of the social and psychological situation. Empirical studies show that individuals will mainly take into account their medical diagnoses and functional status^1^, but also experienced symptoms, medication and other signs of health problems, and particularly in the absence of any evident health problems, different health-related lifestyles and risk factors^6^.

A number of studies have found associations between SRH and biomarker levels (i.e. quantities of biomolecules) in blood, such as white cell count, albumin and haemoglobin^3^, HDL cholesterol^3,7–9^, leptin^10^, TNF-α and IL-1ra^11^, CD19^+^ cells and IgG^12^, CRP^13–15^, IL-6^13,16,17^, fasting plasma glucose and glycosylated haemoglobin^18^ and vitamin D^19,20^. The findings are fragmented, however, as each study usually addresses only a few indicators. Furthermore, most samples have been small, and in many cases other health data have been less than optimal. At least in part, these associations probably reflect the severity and symptoms of chronic diseases. Yet it is possible to hypothesize that the level of and changes in some biomarkers may, through interoceptive processes, be associated with sensations and symptoms that individuals take into account in their self-ratings of health. Overall, little is known about the experiential counterparts of variations in blood biochemistry, although the connections of fatigue with peripheral inflammation for example, are well described^21,22^.

In order to understand the potential of SRH as a measure of health in clinical practice and research, it is important to know how accurately SRH reflects the condition of the human body. In the present explorative study, we investigated the associations of SRH with a wide array of biomolecule levels measured in blood and urine. These biomarkers provide more detailed information about the condition of the human body than diagnostic names alone as they can reflect the stage and severity of current pathologies as well as the physiological processes taking place in individuals without clinical diagnoses. We hypothesize that the possible association between SRH and biomarkers is largely, but not entirely, explained by diseases and physical functioning, which at least to some extent reflects the severity of diseases. We also hypothesize that the association of SRH and mortality is partly explained by the measured biomarkers. We addressed the following questions: (i) to what extent are the biomarkers available in the study associated with SRH; (ii) to what extent are the associations between biomarkers and SRH explained by disease diagnoses and physical functioning; and (iii) do the biomarkers associated with SRH explain part of the association between SRH and mortality? Analyses i) and iii) were also performed for individuals without diagnosed diseases. We used three extensive population-based data sets: MARK-AGE, the Copenhagen Aging and Midlife Biobank (CAMB), and Health 2000, covering a total of approx. 15,000 individuals and 150 biomarkers.

## Results

### Participant characteristics

In MARK-AGE (n = 3,187) 12% rated their health as excellent, 35% as very good, 41% as good, 11% as fair and 1.4% as poor. In CAMB (n = 5,335) the figures were 9.1%, 41%, 40%, 8.6% and 1.4%, respectively. In Health 2000 (n = 6,444) 32% rated their health as good, 30% as rather good, 27% as moderate, 8% as rather poor and 3.5% as poor. Table 1 shows the participants’ characteristics by SRH. In all three data sets poorer SRH was associated with a higher number of diseases and poorer physical functioning (Table 1; Chi-square test: *p* < 0.001).

**Table 1:** Participant characteristics by SRH.

	Self-rated health	MARK-AGE		CAMB		Health 2000	
		*'good', 'very good' or 'excellent'*	*‘poor’ or ‘fair’*	*'good', 'very good' or 'excellent'*	*‘poor’ or ‘fair’*	*'moderate', 'rather good' or 'good'*	*‘poor’ or ‘rather poor’*
**Sample size**							
	% (N)	88 (2798)	12 (389)	90 (4799)	10 (536)	88 (5679)	12 (765)
**Age**							
	Min–Max, years	18–92	35–78	48–62	49–62	30–99	30–97
	Mean (Median), years	57 (59)	62 (64)	54 (56)	54 (56)	52 (49)	65 (67)
**Male**							
	% (N)	88 (1338)	12 (177)	90 (3296)	10 (363)	88 (2554)	12 (349)
**Female**							
	% (N)	87 (1460)	13 (212)	90 (1503)	10 (173)	88 (3125)	12 (416)
**Number of diseases**							
	No diseases, % (N)	95 (1572)	5 (85)	97 (2418)	3 (81)	97 (2489)	3 (85)
	One disease, % (N)	87 (856)	13 (131)	90 (1699)	10 (195)	92 (1777)	8 (163)
	Two diseases, % (N)	73 (280)	28 (106)	79 (564)	21 (153)	79 (882)	21 (232)
	Three diseases, % (N)	62 (83)	39 (52)	58 (102)	42 (74)	71 (397)	29 (160)
	4 + diseases, % (N)	32 (7)	68 (15)	33 (16)	67 (33)	52 (134)	48 (125)
**Difficulties in physical functioning, sum of scores**							
	0, % (N)	98 (485)	2 (10)	98 (1386)	2.2 (31)	97 (1766)	3 (64)
	1 or 2, % (N)	92 (1584)	8 (137)	93 (2915)	7 (210)	95 (2907)	5 (159)
	3 or 4, % (N)	78 (687)	22 (193)	67 (474)	33 (229)	76 (259)	24 (237)
	5 or 6, % (N)	46 (42)	54 (49)	27 (24)	73 (66)	46 (259)	54 (305)

Domain	Concentration of biomarker, unit, mean (median)						
Amino acid metabolism	Alanine minotransferase, U/l	24 (22)	26 (24)	–	–	–	–
Calcium status	Calcium, mmol/l	–	–	–	–	2.4 (2.4)	2.4 (2.4)
Endocrine function	Leptin, ng/l	–	–	–	–	17 (11)	22 (15)
Glucose metabolism	Glucose, mmol/l	5.2 (5.1)	5.7 (5.3)	5.5 (5.3)	5.9 (5.4)	5.5 (5.3)	6.0 (5.5)
	Haemoglobin A1C, %	6.0 (5.9)	6.3 (6.1)	5.3 (5.3)	5.5 (5.5)	5.3 (5.2)	5.7 (5.4)
	Insulin, mU/ml	6.1 (5)	8.5 (5.7)	–	–	9.1 (7.0)	13 (9.0)
Immune system	CRP, mg/l	2.1 (1.2)	3.4 (1.8)	2.2 (1.1)	3.9 (1.9)	2.0 (0.7)	4.0 (1.4)
	CMV antibodies, U/l	39 (22)	53 (37)	–	–	–	–
	Tetanus IgG antibodies, IU/ml	3.7 (1.6)	2.6 (0.78)	–	–	–	–
	Rheumatoid factor, IU/ml	–	–	–	–	19 (15)	30 (15)
Lipid metabolism	Apolipoprotein-A1, g/l	–	–	–	–	1.6 (1.6)	1.6 (1.5)
	Apolipoprotein-B, g/l	–	–	–	–	1.2 (1.2)	1.3 (1.3)
	Clusterin (ApoJClu; Apolipoprotein J/Clusterin in serum), µg/ml	70 (68)	75 (74)	–	–	–	–
	HDL:Total cholesterol, ratio, %	28 (27)	27 (26)	25 (24)	24 (23)	23 (22)	22 (20)
	HDL1 (large particles) Cholesterol, mg/dl	30 (27)	29 (26)	–	–	–	–
	HDL2 (small particles) Cholesterol, mg/dl	38 (38)	37 (36)	–	–	–	–
	HDL2 (small particles) Triglycerides, mg/dl	5.2 (5.0)	5.8 (5.7)	–	–	–	–
	HDL Cholesterol, mol/l ^ELISA^	1.5 (1.5)	1.4 (1.4)	1.5 (1.5)	1.4 (1.3)	1.3 (1.3)	1.3 (1.2)
	HDL Cholesterol, mg/dl ^NMR^	72 (70)	70 (67)	–	–	–	–
	HDL Triglycerides, mg/dl	8.0 (7.6)	9.1 (8.9)	–	–	–	–
	LDL1 (large particles) Cholesterol, mg/dl	59 (58)	50 (50)	–	–	–	–
	LDL2 (small particles) Triglycerides, mg/dl	7.1 (6.3)	7.9 (6.96)	–	–	–	–
	LDL Cholesterol, mmol/l ^ELISA^	3.3 (3.3)	3.2 (3.2)	3.0 (3.0)	2.9 (2.9)	3.7 (3.6)	3.7 (3.7)
	LDL Cholesterol, mg/dl ^MARK-AGE, NMR^	130 (130)	120 (120)	–	–	–	–
	Triglycerides, mg/dl ^MARK-AGE, NMR^ mmol/l ^H2000 & CAMB, ELISA^	110 (94)	130 (110)	1.8 (1.5)	2.0 (1.8)	1.6 (1.3)	1.9 (1.6)
	VLDL1 (large particles) Cholesterol, mg/dl	14 (11)	17 (13)	–	–	–	–
	VLDL1 (large particles) Triglycerides, mg/dl	40 (26)	51 (34)	–	–	–	–
	VLDL2 (small particles) Cholesterol, mg/dl	9.8 (9.1)	10.8 (9.9)	–	–	–	–
	VLDL2 (small particles) Triglycerides, mg/dl	12 (11)	14 (12)	–	–	–	–
	VLDL Cholesterol, mg/dl	26 (22)	30 (25)	–	–	–	–
	VLDL Triglycerides, mg/dl	54 (40)	67 (49)	–	–	–	–
Lipid oxidation product; oxidative stress	8-isoprostane metabolite, in urine, mM/L/mM creatinine	7.3 (6.7)	8.3 (7.3)	–	–	–	–
Marker of tissue damage	Cell-free DNA, µg/ml	0.71 (0.70)	0.74 (0.72)	–	–	–	–
Nutrition	25-OH-VitaminD, nmol/l	52 (49)	44 (41)	–	–	45 (43)	42 (41)
	Alpha-carotene, µmol/l	0.19 (0.14)	0.16 (0.11)	–	–	–	–
	Beta-carotene, µmol/l	0.69 (0.57)	0.50 (0.42)	–	–	–	–
	Beta-cryptoxanthin, µmol/l	0.31 (0.22)	0.22 (0.15)	–	–	–	–
	Hippurate in urine, area (a.u.)	15 (12)	13 (10)	–	–	–	–
	Lutein, µmol/l	0.29 (0.27)	0.24 (0.2)	–	–	–	–
	Normed concentration of vitamin C, pmol/µg DNA	2400 (2100)	2300 (2000)	–	–	–	–
	Retinol in serum, µmol/l	1.8 (1.7)	1.7 (1.7)	–	–	–	–
	Trigonelline in urine, area (a.u.)	1.3 (1.0)	1.2 (0.74)	–	–	–	–
	Zeaxanthin, µmol/l	0.048 (0.043)	0.039 (0.033)	–	–	–	–
Nutrition (alcohol); liver function	Gamma-glutamyltransferase, U/l	21 (14)	28 (16)	–	–	35 (23)	49 (28)
One-carbon metabolism; creatine metabolism	Guanidinoacetate in urine, area (a.u.)	10.7 (8.9)	9.4 (7.2)	–	–	–	–
One-carbon metabolism; CV health	Homocysteine, µmol/l	15 (14)	16 (15)	–	–	12 (11)	15 (13)
Oxidative stress	Protein carbonyls, nmol/mg	0.58 (0.58)	0.59 (0.59)	–	–	–	–
Protein modification	N-glycosylation status of serum proteins, %, peak 9	2.5 (2.4)	2.7 (2.6)	–	–	–	–
	N-glycosylation status of urinary proteins, %, peak 6	3.0 (3.0)	3.4 (3.2)	–	–	–	–
	N-glycosylation status of serum proteins, %, peak 3	7.2 (7.0)	6.9 (6.7)	–	–	–	–
Purine metabolism; anionic form of uric acid	Urate, µmol/l	–	–	–	–	300 (290)	330 (320)
Selenium metabolism	Selenium in plasma, ppb	110 (110)	110 (110)	–	–	–	–
	Plasma Selenium bound to Albumin or Selenoprotein P, ppb	91 (89)	85 (83)	–	–	–	–
	Plasma Selenium bound to Glutathione Peroxidase, ppb	23 (22)	23 (22)	–	–	–	–
Smoking exposure	Cotinine, µg/l	–	–	–	–	120 (10)	130 (9)
Tissue repair	Fibrinogen, mg/ml	3.6 (3.4)	4.0 (3.8)	–	–	–	–
Renal function	Albumin in urine, mg/l	–	–	–	–	14 (3.1)	45 (4.3)

The 57 biomarkers presented in the table were all associated with SRH in linear modelling when adjusted for age and gender (model i).

*a.u.* arbitrary unit, *CMV*  cytomegalovirus, *CRP*  C-reactive protein, *CV*  cardiovascular, *ELISA*  enzyme-linked immunosorbent assay, *HDL*  high-density lipoprotein, *IU*  international unit, *IL*  interleukin, *LDL*  low-density lipoprotein, *NMR*  nuclear magnetic resonance, *OH*  hydroxyl, *ppb*  parts per billion, *VLDL*  very low-density lipoprotein.

### Biomarkers associated with SRH

**Figure 1:**
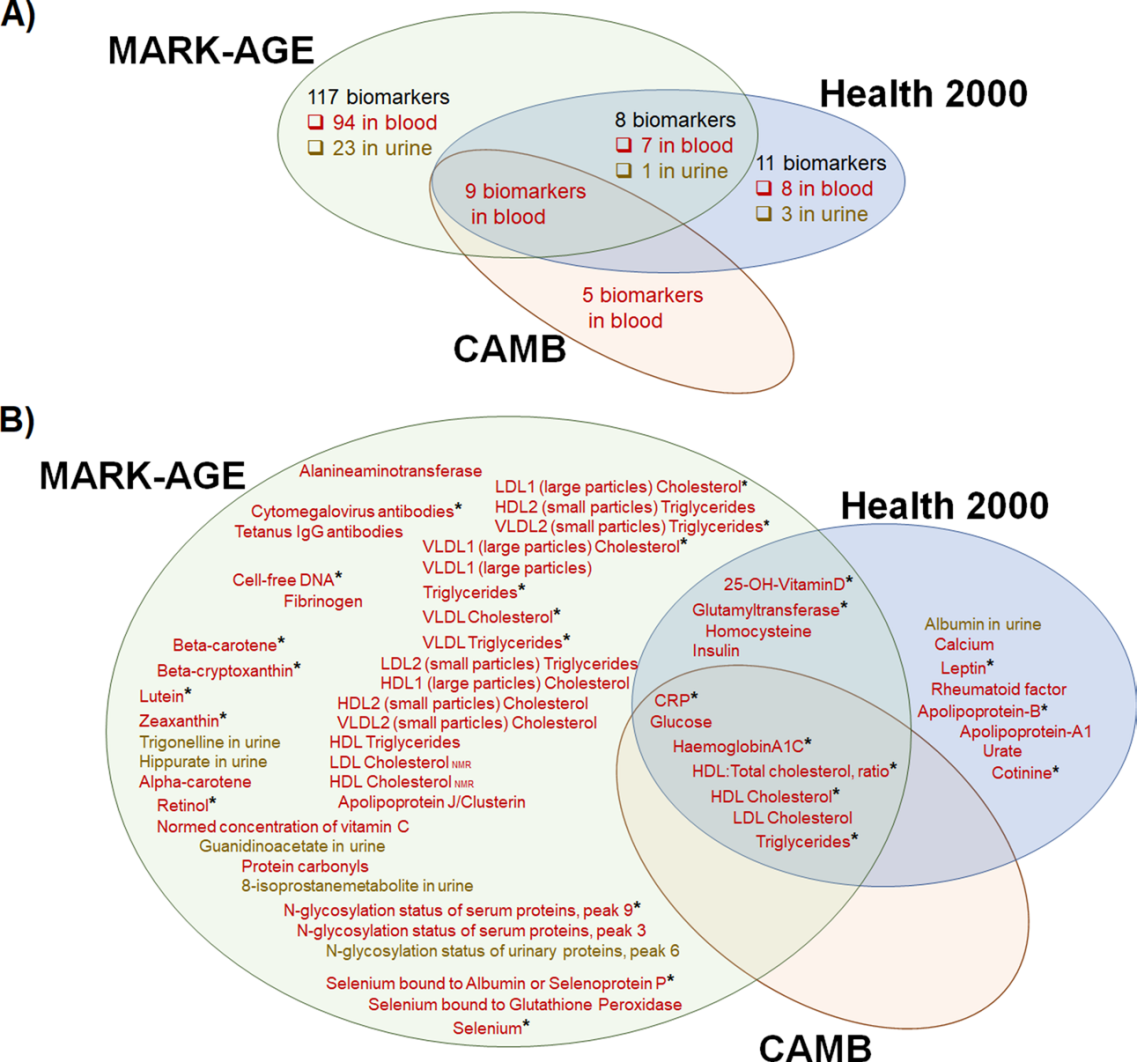
A summary of (**A**) the number of biomarkers in the analysis in the three data sets and (**B)** biomarkers associated with SRH, adjusted for age and gender (Model i, in the full samples). Sample types are indicated with colors: red = blood, yellow = urine, and * symbol indicates that association was significant also after the adjustment for diseases and physical functioning (Model ii, in the full samples).

Out of the 150 biomarkers investigated (Fig. 1A, Supplementary information 1, Supplementary information 2: Table S1 and S2), 57 were significantly associated with SRH in linear regression analyses that were adjusted for age and gender (model i). Table 1 shows the means and medians by SRH for these 57 biomarkers. Eleven of them were available in two or three data sets (Table 2, Supplementary information 2: Table S1 and S2) and they showed similar directions of associations across the data sets. Of the 57 biomarkers, 46 were available for analysis in one data set only (Table 3, Supplementary information 2: Table S1 and S2).

**Table 2:** Associations between biomarkers and SRH in linear regression analysis, adjusted for (i) age and gender and (ii) additionally for number of diseases and physical functioning.

Domain	Biomarker	MARK-AGE				CAMB				Health 2000			
		β_i_	p_i_	+ physical functioning and number of diseases		β_i_	p_i_	+ physical functioning and number of diseases		β_i_	p_i_	+ physical functioning and number of diseases	
				β_ii_	p_ii_			β_ii_	p_ii_			β_ii_	p_ii_
Muscle metabolism; renal health	Creatinine	− 0.0011	0.24	− 0.0012	0.15	–	–	–	–	− 0.00062	0.50	− 0.0016	**0.046**
	Creatinine in urine	0.00091	0.75	0.0030	0.24	–	–	–	–	− 0.00079	0.74	0.00084	0.69
Glucose and lipid metabolism	Adiponectin	− 0.0058	**0.012**	− 0.0028	0.18	–	–	–	–	− 0.0016	0.53	− 0.00050	0.82
Glucose metabolism	Insulin	0.014	** < 0.001**	0.0059	**0.003**	–	–	–	–	0.0011	**0.007**	0.00036	0.32
	Glucose	0.089	** < 0.001**	0.043	** < 0.001**	0.047	** < 0.001**	0.0092	0.17	0.063	** < 0.001**	0.015	0.086
	Haemoglobin A1C, %	0.18	** < 0.001**	0.080	** < 0.001**	0.21	** < 0.001**	0.072	** < 0.001**	0.18	** < 0.001**	0.075	** < 0.001**
Iron metabolism	Ferritin	0.00025	0.18	0.00010	0.54	–	–	–	–	0.00055	** < 0.001**	0.00041	** < 0.001**
Lipid metabolism	HDL:Total cholesterol	− 0.011	** < 0.001**	− 0.0080	** < 0.001**	− 0.0089	** < 0.001**	− 0.0068	** < 0.001**	− 0.0098	** < 0.001**	− 0.0069	** < 0.001**
	HDL Cholesterol	− 0.33	** < 0.001**	− 0.16	** < 0.001**	− 0.27	** < 0.001**	− 0.16	** < 0.001**	− 0.24	** < 0.001**	− 0.10	**0.001**
	Cholesterol, total	− 0.074	** < 0.001**	− 0.015	0.31	− 0.034	** < 0.001**	− 0.0045	0.60	− 0.015	0.17	0.023	**0.018**
	LDL Cholesterol	− 0.062	** < 0.001**	− 0.010	0.55	− 0.027	**0.039**	0.0080	0.49	− 0.029	**0.014**	0.017	0.11
	Triglycerides	0.0020	** < 0.001**	0.0011	** < 0.001**	0.092	** < 0.001**	0.044	** < 0.001**	0.10	** < 0.001**	0.054	** < 0.001**
Nutrition; UVB exposure	25-OH-VitaminD	− 0.0052	** < 0.001**	− 0.0040	** < 0.001**	–	–	–	–	− 0.0064	** < 0.001**	− 0.0026	** < 0.001**
Nutrition (alcohol problem); liver function	Glutamyl transferase	0.0022	** < 0.001**	0.0013	**0.009**	–	–	–	–	0.0022	** < 0.001**	0.0016	** < 0.001**
One-carbon metabolism; CV health	Homocysteine	0.0070	**0.008**	0.0026	0.28	–	–	–	–	0.018	** < 0.001**	0.0056	**0.019**
Immune system	CRP	0.035	** < 0.001**	0.020	** < 0.001**	0.028	** < 0.001**	0.011	** < 0.001**	0.016	** < 0.001**	0.0053	**0.004**
Oxygen transfer	Haemoglobin	− 0.0052	0.30	− 0.0028	0.52	0.044	**0.012**	0.014	0.35	0.0011	0.27	0.0018	**0.039**

*CRP*  C-reactive protein, *CV* cardiovascular, *HDL* high-density lipoprotein, *IU* international unit, *IL* interleukin, *LDL* low-density lipoprotein, *OH*  hydroxyl, *UVB* ultraviolet B light, *VLDL*  very low-density lipoprotein.

This table presents the results for all biomarkers (n = 17) available in two or three data sets (MARK-AGE, CAMB, and/or Health 2000). Criteria for significant association of a biomarker was *p* < 0.05 in all data sets where it was analysed. p-values less than 0.05 are shown in bold. Results for all biomarkers are shown in Supplementary information 1.

**Table 3:** Associations between biomarkers and SRH in linear regression analysis, adjusted for (i) age and gender and (ii) additionally for number of diseases and physical functioning.

Domain	Biomarker	Data set	β_i_	p_i_	+ physical functioning and number of diseases	
					**β**_**ii**_	**p**_**ii**_
Selenium metabolism	Selenium	MARK-AGE	− 0.00564	**8.28 × 10**^**–16**^	− 0.00433	**9.24 × 10**^**–12**^
Selenium metabolism	Selenium bound to Albumin or Selenoprotein P	MARK-AGE	− 0.00645	**5.84 × 10**^**–16**^	− 0.00490	**1.15 × 10**^**–11**^
Smoking exposure	Cotinine	Health 2000	0.000382	**7.33 × 10**^**–14**^	0.000294	**1.41 × 10**^**–10**^
Nutrition	Lutein	MARK-AGE	− 0.663	**2.12 × 10**^**–12**^	− 0.526	**5.88 × 10**^**–10**^
Nutrition	Beta-cryptoxanthin	MARK-AGE	− 0.421	**7.01 × 10**^**–14**^	− 0.308	**1.27 × 10**^**–09**^
Endocrine function	Leptin	Health 2000	0.00892	**1.82 × 10**^**–26**^	0.00375	**1.11 × 10**^**–06**^
Nutrition	Beta-carotene	MARK-AGE	− 0.251	**2.39 × 10**^**–16**^	− 0.135	**1.20 × 10**^**–06**^
Lipid metabolism	Apolipoprotein-B	Health 2000	0.177	**4.02 × 10**^**–05**^	0.180	**3.10 × 10**^**–06**^
Lipid metabolism	VLDL1 (large particles) Cholesterol	MARK-AGE	0.0110	**1.81 × 10**^**–12**^	0.00658	**3.44 × 10**^**–06**^
Nutrition	Zeaxanthin	MARK-AGE	− 3.42	**1.06 × 10**^**–11**^	− 2.06	**5.98 × 10**^**–06**^
Lipid metabolism	VLDL Triglycerides	MARK-AGE	0.00242	**3.65 × 10**^**–13**^	0.00132	**1.39 × 10**^**–05**^
Lipid metabolism	VLDL1 (large particles) Triglycerides	MARK-AGE	0.00253	**1.45 × 10**^**–12**^	0.00138	**2.15 × 10**^**–05**^
Lipid metabolism	VLDL Cholesterol	MARK-AGE	0.00686	**2.93 × 10**^**–11**^	0.00394	**2.47 × 10**^**–05**^
Lipid metabolism	VLDL2 (small particles) Triglycerides	MARK-AGE	0.0229	**3.60 × 10**^**–13**^	0.0119	**3.58 × 10**^**–05**^
Protein modification	N-glycosylation status of serum proteins, peak 9	MARK-AGE	0.0577	**1.26 × 10**^**–05**^	0.0462	**9.70 × 10**^**–05**^
Tissue damage	Cell-free DNA	MARK-AGE	0.693	**2.08 × 10**^**–07**^	0.464	**9.99 × 10**^**–05**^
Lipid metabolism	LDL1 (large particles) Cholesterol	MARK-AGE	− 0.00515	**2.13 × 10**^**–15**^	− 0.00233	**0.000108**
Nutrition	Retinol	MARK-AGE	− 0.145	**8.15 × 10**^**–05**^	− 0.126	**0.000154**
Immune system	Cytomegalovirus antibodies	MARK-AGE	0.00182	**1.05 × 10**^**–07**^	0.00113	**0.000235**
Purine metabolism	Urate	Health 2000	0.00129	**5.02 × 10**^**–14**^	0.000205	0.190
Lipid metabolism	HDL2 (small particles) Triglycerides	MARK-AGE	0.0570	**3.16 × 10**^**–13**^	0.0240	0.000805
Lipid metabolism	HDL Cholesterol, NMR	MARK-AGE	− 0.00561	**8.12 × 10**^**–09**^	− 0.00285	0.00125
Nutrition	Trigonelline in urine	MARK-AGE	− 0.0913	**8.78 × 10**^**–09**^	− 0.0469	0.00112
Creatine metabolism	Guanidinoacetate (3–97s) in urine	MARK-AGE	− 0.0139	**2.39 × 10**^**–08**^	− 0.00604	0.00753
Nutrition	Hippurate in urine	MARK-AGE	− 0.00894	**2.61 × 10**^**–08**^	− 0.00396	0.00663
Lipid metabolism	LDL2 (small particles) Triglycerides	MARK-AGE	0.0229	**1.84 × 10**^**–07**^	0.0126	0.00148
Lipid metabolism	HDL1 (large particles) Cholesterol	MARK-AGE	− 0.00592	**3.45 × 10**^**–07**^	− 0.00283	0.00715
Lipid metabolism	HDL2 (small particles) Cholesterol	MARK-AGE	− 0.0129	**6.50 × 10**^**–07**^	− 0.00731	0.00174
Lipid metabolism	LDL Cholesterol, NMR	MARK-AGE	− 0.00243	**2.26 × 10**^**–06**^	− 0.000635	0.176
Transporter protein	Albumin in urine	Health 2000	0.000491	**5.83 × 10**^**–06**^	0.0000643	0.512
Immune system	Tetanus IgG antibodies	MARK-AGE	− 0.0132	**6.35 × 10**^**–06**^	− 0.00871	0.000904
Lipid metabolism	VLDL2 (small particles) Cholesterol	MARK-AGE	0.0171	**6.59 × 10**^**–06**^	0.0103	0.00254
Aminoacid metabolism	Alanineaminotransferase	MARK-AGE	0.00685	**7.76 × 10**^**–06**^	0.00337	0.0149
Calcium status	Calcium	Health2000	0.652	**1.21 × 10**^**–05**^	0.210	0.118
Lipid metabolism	HDL Triglycerides	MARK-AGE	0.0169	**1.32 × 10**^**–05**^	0.00684	0.0504
Protein modification	N-glycosylation status of serum proteins, peak 3	MARK-AGE	− 0.0424	**2.35 × 10**^**–05**^	− 0.0261	0.0039
Oxidative stress	Proteincarbonyls	MARK-AGE	0.704	**2.94 × 10**^**–05**^	0.537	0.0004
Nutrition	Alpha-carotene	MARK-AGE	− 0.333	**3.03 × 10**^**–05**^	− 0.0743	0.304
Tissue repair and revascularization	Fibrinogen	MARK-AGE	0.0469	**3.86 × 10**^**–05**^	0.0256	0.0125
Selenium metabolism	Selenium bound to Glutathione Peroxidase	MARK-AGE	− 0.0117	**4.82 × 10**^**–05**^	− 0.00891	0.000565
Lipid metabolism	Apolipoprotein-A1	Health 2000	− 0.177	**4.90 × 10**^**–05**^	− 0.0469	0.231
Protein modification	N-glycosylation status of urinary proteins, peak 6	MARK-AGE	0.0343	**8.28 × 10**^**–05**^	0.0254	0.00119
Lipid metabolism	Apolipoprotein J/Clusterin	MARK-AGE	0.00310	**9.39 × 10**^**–05**^	0.00159	0.0264
Lipid oxidation product	8-isoprostanemetabolite, in urine	MARK-AGE	0.0184	**0.000104**	0.00804	0.0600
Immune system	Rheumatoid factor	Health 2000	0.000820	**0.000126**	0.000447	0.0200
Nutrition	Normed concentration of vitamin C	MARK-AGE	− 0.0000410	**0.000169**	− 0.0000231	0.0183

*CRP* C-reactive protein, *HDL* high-density lipoprotein, *LDL* low-density lipoprotein, *NMR* nuclear magnetic resonance, *VLDL* very low-density lipoprotein.

The Table shows 46 biomarkers that were available for analysis in one data set only and were associated with SRH in Model (i). 19 of them were associated with SRH also in Model (ii). The results for all biomarkers are shown in Supplementary information 1. Statistical significance level was at Bonferroni-adjusted *p* value of 0.05, and *p* values below this threshold are shown in bold.

When additionally adjusted for number of diseases and physical functioning (model ii), 26 biomarkers were still associated with SRH. Seven of these biomarkers were available in two or three data sets (Table 2) and 19 biomarkers in one data set only (Table 3). A schema of the analysis pipeline and summaries of the findings are shown in Supplementary information 2 (Figure S1, Table S1 and S2) and Fig. 1B.

**Figure 2:**
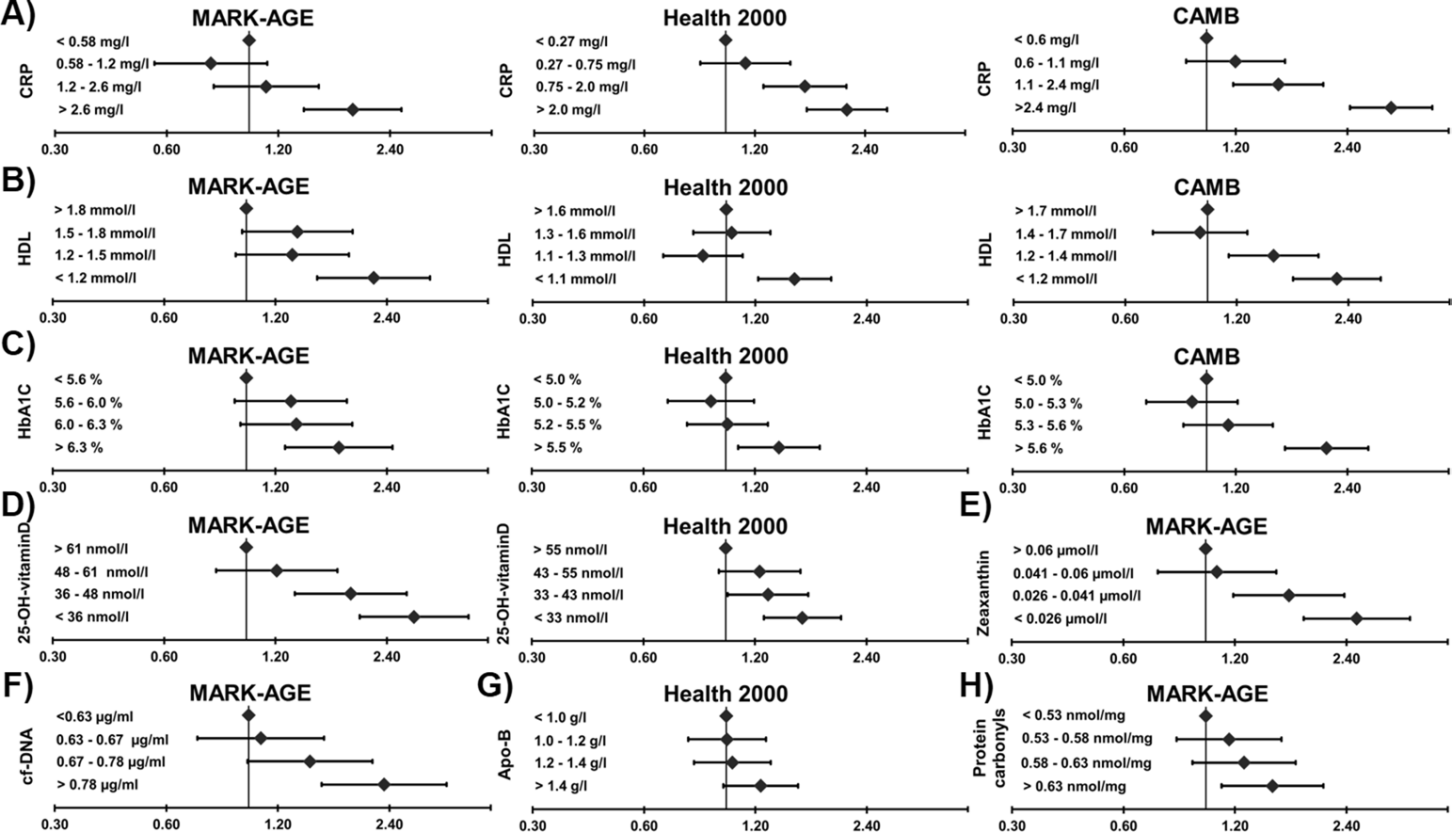
Associations of the eight selected biomarkers, categorized as quartiles, with poor self-rated health. Odds ratios (ORs) and 95% confidence intervals (CIs) are from the logistic regression models, adjusted for age and gender. The “best” biomarker quartile was used as reference category. (**A**–**H**): C-reactive protein (CRP), high-density lipoprotein (HDL) cholesterol, haemoglobin A1C (HBA1C), 25-hydroxyl-vitamin-D (25-OH-vitaminD), zeaxanthin, apolipoprotein-B (Apo-B), cell-free DNA (cf-DNA) and protein carbonyls, respectively. The biomarkers were selected as examples of replicated and new associations representing different biological domains.

As examples of replicated and new findings representing different biological domains, Fig. 2 shows the associations of eight selected biomarkers, categorized as quartiles, with SRH. Most of these analyses showed a graded association of poorer SRH with poorer biomarker levels.

### Mortality analysis

In the Health 2000 data the association between SRH and mortality (iii) was analysed using Cox regression analysis. Out of 5,957 participants with a full set of data for relevant variables, 1,207 (20%) died between 2000 and 2015. The average survival time for the deceased participants was 8.1 (SD 4.2) years.

**Figure 3:**
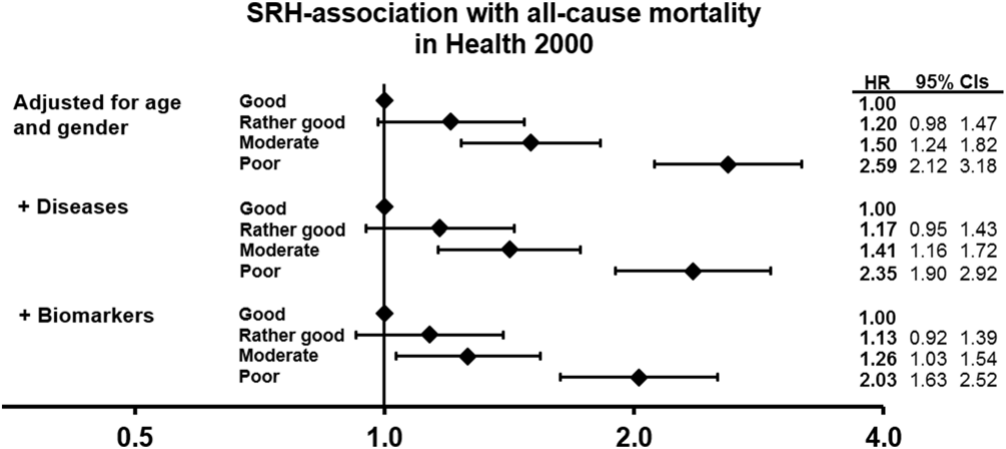
Association of self-rated health with mortality in the Health 2000 data (n = 5,957; deceased n = 1,207; 15-year follow-up). In Cox proportional hazards models (iii) hazard ratios (HRs) and 95% CIs were adjusted for (1) age and gender; (2) then additionally for number of diseases; and then (3) additionally for 10 biomarkers.

A graded relationship was found between SRH and mortality in models adjusting for age and gender, and then additionally for diseases (Fig. 3). In the third model the analysis was further adjusted for ten biomarkers that were significantly associated with SRH (model ii, results shown in Tables 2 and 3: leptin, apolipoprotein B, cotinine, HbA1C, HDL:total cholesterol ratio, HDL cholesterol, triglycerides, 25-hydroxy-vitaminD, gamma-glutamyltransferase and CRP). The association between SRH and mortality was weakened after the addition of these biomarkers to the model, but it still remained significant: hazard ratios (95% CIs) 1.1 (0.9, 1.4), 1.3 (1.0, 1.5) and 2.0 (1.6, 2.5), respectively.

### Analyses for individuals without diseases

All analyses were also conducted separately for subsamples of individuals who had no disease diagnoses (MARK-AGE n = 1657, CAMB n = 2499, Health 2000 n = 2574). Descriptive statistics for these participants are shown in Supplementary information 2: Table S3. In linear regression models adjusted for age and gender, 8 biomarkers that were available for analysis in two or three data sets (*p* < 0.05 in all data sets ) and 8 biomarkers that were available in one data set (Bonferroni-adjusted *p* value < 0.05) were significantly associated with SRH (Supplementary information 2: Table S1, S4 and S5). As in the results for the whole data, CRP, triglycerides and HDL cholesterol showed a significant association in all three data sets, and vitamin D in the two data sets where it was available. For the biomarkers that in the full data were significantly associated with SRH, with only few exceptions, the direction of the associations was the same among the “healthy” participants, although the associations in the latter group did not always reach statistical significance. Results for all biomarkers from the analysis in healthy individuals are shown in Supplementary information 3.

For the subsample of individuals without diagnoses (n = 2,408) in the Health 2000 data, the association between SRH and mortality was analysed using Cox regression analysis. During the 15-year follow-up the number of deaths was 193 (8%), and average survival time for the deceased participants in this sample was 9.2 (SD 4.0) years. In this subsample SRH was a significant dose-responsive predictor of mortality (Supplementary information 2: Figure S2). When the biomarkers that showed a significant association with SRH in the full data analysis (Table 2 and 3, model (ii) were included in the model, and when good SRH was set as the reference category, hazard ratios (95% CIs) for rather good, moderate and poor SHR were 1.2 (0.9, 1.7), 1.3 (0.9, 1.9) and 2.3 (1.3, 3.8), respectively.

## Discussion

The underlying assumption of this work was that SRH is a more comprehensive and sensitive indicator of the condition of the human organism than medical diagnoses or measures of physical functioning alone. We hypothesized that if this was true, SRH should show an association with blood and urine biomarkers that reflect the physiological regulation of the organism. Therefore, we analysed 150 biomarkers from almost 15,000 participants enrolled in three population-based studies. Altogether 57 biomarkers showed a significant association with SRH, and for 26 of them the association was upheld when the number of chronic diseases and physical functioning were taken into account. In subsamples of individuals without chronic diseases, 16 biomarkers were associated with SRH. These associations were almost exclusively in a logical direction, i.e. a “worse” biomarker level was associated with poorer SRH and vice versa. Moreover, biomarkers weakened the association between SRH and mortality.

We had no a priori hypothesis as to which biomarkers are important regarding SRH. In this explorative study we included all blood and urine measures that were available in the study samples. Our results confirm the previous evidence for most biomarkers that have been reported to be associated with SRH, and they additionally reveal a large number of new associations. These biomarkers are descriptive of various biological systems of the human body, including inflammation (e.g. CRP), lipid and glucose metabolisms (e.g. cholesterol and HbA1C), oxidative stress (e.g. protein carbonyls) and tissue damage (cell-free DNA), as well as of lifestyles and environmental exposures (e.g. carotenoids, vitamin D, cotinine). Many of the biomarkers associated with SRH are also known to be biomarkers of ageing^23^.

When selected biomarkers—CRP, HDL cholesterol, HbA1C, 25-hydroxyl-vitamin-D, zeaxanthin, apolipoprotein-B, cell-free DNA and protein carbonyls—were picked up as examples and examined as quartiles, poorer biomarker levels were fairly constantly associated with higher odds for poorer SRH in all data sets (Fig. 2). CRP is a proinflammatory marker and known to be associated with SRH^13–15^. A few studies have also reported an association between poorer SRH and lower HDL cholesterol^3,7–9^ and higher HbA1C^18^ levels. In our study lower vitamin D level was associated with poorer SRH in both data sets where it was available, and a similar, strong association was also seen among individuals without chronic conditions. Previous studies with smaller samples have likewise shown an association between vitamin D and SRH^19,20^. Recent studies have connected low vitamin D concentration with multiple extra-skeletal processes such as cancer progression, coronary heart disease, depression and a range of immune functions^24–26^. The mechanisms of these associations are not well known, but it has been suggested that low vitamin D level should be understood as a marker of ill health rather than a causal factor^27,28^.

This is the first study to report associations between SRH and e.g. zeaxanthin, apolipoprotein-B, cell-free DNA and protein carbonyls. Zeaxanthin is a carotenoid pigment present in the eye and obtained from the diet (e.g. egg yolk and orange peppers)^29^. It has antioxidative properties and is suggested to have a protective role against eye diseases (especially age-related macular degeneration) as well as cardiovascular diseases and cancer^30^. Apolipoprotein B is mostly known as the LDL carrier protein, and it is an important contributor to atherosclerosis and cardiovascular disease^31^. Circulating cell-free DNA is a marker of cellular death and tissue damage in many acute and chronic conditions (e.g. sepsis, trauma, aseptic inflammation, cardiovascular diseases and cancer)^32–37^. Elevated levels of protein carbonyls (i.e. plasma protein oxidation levels) are a marker of oxidative stress and observed in various pathologies such as Alzheimer’s disease, rheumatoid arthritis, diabetes, sepsis, renal dysfunction and respiratory failure^38^.

We suggest that there are three main pathways through which biomarkers measured in blood or urine can affect SRH. First, several biomarkers are characteristics of clinical diagnoses. For certain biomarkers such as cholesterol or glucose levels the role is well-known as is their significance as risk factors of disease. Individuals who are asked to rate their own health may be inclined to interpret high values (if they know them) as signs of poorer health. Yet respondents may not necessarily consider their biomarker levels or even be aware of them when asked to assess their health, but instead consider their disease diagnoses, symptoms or decreased physical functioning caused by their diseases. In this case the association between biomarkers and SRH is indirect and mediated by the association of SRH with diseases known to the respondents. This hypothesis for disease pathway is supported by the finding that associations between biomarkers and SRH were more marked among individuals with disease than those without.

Second, it is known that particularly individuals without major health problems take account of health-related lifestyles and behavioural risk factors as components of SRH^6,39^. In the present study better SRH was associated with higher levels of carotenoids (zeaxanthin, beta-carotene, lutein, beta-cryptoxanthin and beta-carotene) in plasma, and hippurate and trigonelline in urine. These molecules serve as markers of fruit and vegetable intake. Worse SRH was associated with higher cotinine and gamma-glutamyltransferase levels, which serve as markers for smoking exposure and alcohol consumption, respectively. Again it is plausible that the route from biomarkers to SRH is indirect, i.e. that respondents assess their health as good or poor not on the basis of their biomarker levels but rather particular health-related lifestyles that are considered healthy or unhealthy.

Third, an interesting but poorly understood mechanism is the possibility that a biomarker level or change in biomarker level in the body might stimulate physical sensations, and that these sensations are interpreted as information about the state of one’s health. This is not a novel hypothesis but was suggested by Stenback as early as 1964 and later by Kaplan and Camacho in 1983 as one potential explanation for the association between SRH and mortality^40,41^. Since these studies, research has continued to accumulate about the interoceptive processes through which information on internal states of the body is communicated to the brain to enable the regulation of vital inner processes and the maintenance of physiological stability^21,42–44^.Most of the research data on interoceptive signalling of humoral processes, i.e. changes in blood substance levels, concerns inflammation: higher circulating levels of inflammatory biomarkers, cytokines, are known to underlie symptoms such as fatigue, general malaise, poor appetite and low mood^21,22^, and they are known to be associated with poor SRH^45,46^. In our study, higher levels of inflammatory markers such as CRP and IL-18 showed associations with poor SRH, and for CRP this was true in all three data sets independently of diseases and physical functioning. Yet the empirical evidence on interoceptive signalling of humoral processes remains haphazard and for other blood-measured substances than inflammatory markers almost non-existent.

In our study, as in many previous ones, SRH showed a strong, robust association with mortality. Poorer SRH predicted mortality even after adjusting for chronic conditions in the total sample and in the subsample without chronic conditions. Adjusting for biomarkers weakened this association in both situations, which supports our initial hypothesis.

We were able to utilize three large population-based data sets from multiple European countries, but, unfortunately, not all biomarkers were available in more than one data set, which would allowed the principles of conventional replication studies. For some of the biomarkers investigated the association with SRH had already been reported earlier. The strengths of our study included the fact that we had access to a large number of new biomarkers; that several of them were available in more than one data set; that we had access to data on chronic conditions and physical functioning; and that in one sample it was also possible to investigate mortality. Multimorbidity indicated by the number of disease diagnoses is an effective descriptive of health and has prognostic value^47–49^. In our analysis, the six clinical diagnoses available for analysis in the three study samples were common and chronic. However, several important diagnostic categories were absent from our analysis, and we had no information on disease severity other than physical functioning. Therefore, the data available was not ideal to adjust for disease. These are the major limitations of the study. In our exploratory analyses, linear regression models were used to provide an easily understandable overview on the associations of SRH, basically an ordinal variable, with biomarkers; this approach is consistent with several earlier studies, and based on observed continuity in its association with many other health variables. Further, because of the explorative approach, we decided not to construct organ-specific or cluster-based groupings of the biomarkers, and as the role of individual biomarkers in connection to SRH is not known, we decided to include all available measures in our analyses without selection. The mechanisms linking biomarkers with SRH were also beyond the scope of this study. Further studies are needed to shed light on the full pathways between SRH and the biological state of the body.

In conclusion, our study demonstrated strong and logical associations of SRH with numerous biomarkers measured in blood and urine, even independently of chronic diseases and functional status. Poorer SRH was associated with worse biomarker levels and vice versa. These biomarkers were descriptive of many different organ systems and bodily processes. The findings suggest that SRH has a solid biological basis. Our results also lend support to the notion that SRH is a robust, comprehensive but non-specific indicator that can more exhaustively capture health-related processes than many conventional measures of health and disease. To verify the potential of SRH in research and in clinical practice, multidisciplinary research is needed to explore the mechanisms that convey messages from body biology to individuals’ subjective assessments.

## Methods

### Study populations

In *MARK-AGE*, questionnaires and interviews were conducted and biological data collected between 2008 and 2012 at the following recruiting centres: Hall in Tyrol/Innsbruck (Austria), Namur (Belgium), Esslingen (Germany), Athens and surrounding regions (Greece), Bologna (Italy), Warsaw (Poland), Tampere (Finland) and Leiden (The Netherlands)^50,51^. The total number of participants in this analysis was 3,187 (age range 18–92 years).

*CAMB* collected questionnaire data and biological samples from 5,335 participants (age range 48–62 years) in 2009–2011. This data set comprises participants from three cohort studies: the Metropolit 1953 Danish Male Birth Cohort (MP), the Copenhagen Perinatal Cohort (CPC) born in 1959–1961, and the Danish Longitudinal Study on Work, Unemployment and Health (DALWUH) born in 1949 or 1959^52^.

*Health 2000* is a nationwide survey conducted in 2000–2001 with a randomly selected sample (n = 8,028) of the Finnish population aged 30 years or over^53^. For this analysis we used a subsample of 6,444 participants (age range 30– 99 years) with relevant information.

No human participants were directly involved in the current study and only data was taken for the current study.

### Measures

SRH was assessed in interviews and questionnaires. In MARK-AGE and CAMB, SRH was inquired by asking: “In general, would you say your health is…?”; and in Health 2000 by asking: “Is your present state of health…?”. The response options were “poor”, “fair”, “good”, “very good” or “excellent” (CAMB and MARK-AGE) and “poor”, “rather poor”, “moderate”, “rather good” or “good” (Health 2000). In linear regression models, SRH was used as a continuous variable ranging from 0 to 4, with a higher value referring to poorer SRH. For the other analyses, the two poorest SRH categories (poor & fair in MARK-AGE and CAMB; poor & rather poor in Health 2000) were combined into one. Then, as an outcome in logistic regression analyses (in Fig. 2), SRH was dichotomized as poor versus all other categories. In mortality analysis (Health 2000) SRH was grouped into four categories: (1) good, (2) rather good, (3) moderate and (4) poor.

A total of 150 biomarkers measured in blood and urine were available for analysis (full list shown in Supplementary information 1): 134 biomarkers in MARK-AGE, 14 in CAMB, and 28 in Health 2000. All measurements were carried out in accordance with relevant guidelines and regulations. Altogether 17 biomarkers were available in two or three of the study populations. A few biomarkers were measured with two different but equivalent measurements. The proportion of missing biomarker data ranged from 0.05 to 25%. A few biomarkers representing different biological domains were selected for inclusion in Fig. 2 as examples of previously shown and new associations with SRH. For this illustration, biomarker levels were categorized as quartiles.

The indicator of physical functioning came from interviews, questionnaires and hand grip strength measurements. A summary variable for physical functioning was constructed out of three components: (1) *ability to walk* 0.5 mile (in MARK-AGE), 0.25 mile (in CAMB) or 0.5 km (in Health 2000); (2) *ability to run 100 m* (in CAMB and Health 2000) or *do vigorous activities* such as running, lifting heavy objects, participating in strenuous sports (in MARK-AGE); and (3) *hand grip strength* (MARK-AGE, CAMB, Health 2000). In each of these three components, more points corresponded to poorer functioning. The components of *walking* and *running & vigorous activities* were scored as 0 = no limitations, 1 = moderate limitations and 2 = highly limited or cannot do at all. *Hand grip strength* was grouped in tertiles (categories 0, 1 and 2). The scores from the three components were added together to obtain a sum score of physical functioning, ranging from 0 to 6.

Disease diagnoses, including cardiovascular diseases, hypertension, diabetes, cancer/tumour, respiratory diseases and arthritis were obtained from interview and questionnaire data. The variable “number of diseases” ranged from 0 to 6 diseases, but in the final analyses it was categorized as 0, 1, 2, 3 or 4 + diseases. In addition, subsamples with participants without any of the above mentioned diagnoses were extracted in each data set (MARK-AGE n = 1,657; CAMB n = 2,499; Health 2000 n = 2574; characteristics in Supplementary information 2: Table S3).

Mortality data were only available for the Health 2000 sample. Dates of death were drawn from the National Register on Causes of Death maintained by Statistics Finland, and the length of follow-up was 15 years.

### Statistical analysis

The association between each individual biomarker and SRH was first explored using linear regression analysis in the three independent cross-sectional data sets (MARK-AGE, CAMB and Health 2000). SRH was the dependent variable, and the models were adjusted for (i) age as a continuous variable and gender, and (ii) additionally for the number of diseases and physical functioning. All MARK-AGE analyses were adjusted for recruitment centre. The nominal p-value threshold was set at 0.05 for biomarkers that were available in two or three data sets (specifically, it was required that the p-value threshold had to be met in all data sets), and, to control the multiple testing problem, at Bonferroni-adjusted p-value of 0.05 for biomarkers that were available in one data set only. Additionally, logistic regression models were used to analyse the associations of eight selected biomarkers, categorized as quartiles, with poor SRH, adjusted for age and gender. The results of the eight selected biomarkers were visualized as forest plots (Fig. 2).

Next, the association of SRH with all-cause mortality in the Health 2000 data set was analysed using Cox proportional hazard modelling (iii). 1) The model was adjusted for (1) age and gender; (2) additionally for number of diseases; and (3) furthermore additionally for the biomarkers that were associated with SRH in the linear regression analysis (Model ii). The nominal p-value threshold was set at 0.05. These results were visualized as forest plot (Fig. 3).

Finally, we repeated the analyses of the associations between SRH and the biomarkers in all three data sets and the mortality analysis in the Health 2000 data in subsamples without disease diagnoses. The criteria for statistical significance were the same as in the main analysis.

The data was processed, analysed and visualized using R software (R 3.4.0) and IBM SPSS software version 24.0 (IBM Corp., Armonk, New York, USA). In each model, participants with missing data for a biomarker, mortality, age, gender, SRH, physical functioning or number of diseases were excluded from the analyses.

### Ethics approval

Human participants were not directly involved in the current study and only existing data was taken for the current analysis. The study was conducted in accordance with the Declaration of Helsinki ethical principles and all research participants gave their informed consent to be part of the study. The studies (MARK-AGE, CAMB, Health 2000) were approved by the local ethics committees^51–53^.

## Supplementary Information

Supplementary Information 1.

Supplementary Information 2.

Supplementary Information 3.

## Data Availability

The data used in the current study are not publicly available for ethical reasons. However, data are available upon request from the Health 2000 survey, MARK-AGE and CAMB for researchers who meet the criteria for access to confidential data. Data from the MARK-AGE study are available from the MARK-AGE steering committee (contact: Alexander Bürkle, alexander.buerkle@uni-konstanz.de). Data from CAMB are available from the Copenhagen Aging and Midlife Biobank steering committee (https://camb.ku.dk/, contact: Rikke Lund, rilu@sund.ku.dk). Health 2000 data are available from THL on request, subject to the submission of approved study proposals and a data transfer agreement (contact: terveys-2000-2011@thl.fi).
