# Supplementary material for: Self-rated health in individuals with and without disease is associated with multiple biomarkers representing multiple biological domains
Source: Sci Rep. 2021 Mar 17;11:6139. doi: 10.1038/s41598-021-85668-7 (PMC7969614; doi:10.1038/s41598-021-85668-7)
Supplement: Supplementary file 2 — Supplementary Information 2. [file 41598_2021_85668_MOESM2_ESM.docx]

**Self-rated health in individuals with and without disease is associated with multiple biomarkers representing multiple biological domains**

L. Kananen^1,2,3^, L. Enroth^1,2^, J. Raitanen^1,2^, J. Jylhävä^2,4^, A. Bürkle^5^, M. Moreno-Villanueva^5^, J. Bernhardt ^6^, †O. Toussaint^7^, B. Grubeck-Loebenstein^8^, M. Malavolta^9^, A. Basso^9^, F. Piacenza^9^, S. Collino^10^, ES. Gonos^11^, E. Sikora^12^, D. Gradinaru^13^, EHJM. Jansen^14^, MET. Dollé^14^, M. Salmon^15^, W. Stuetz^16^, D. Weber^17^, T. Grune^17,18,19^, N. Breusing^19^, A. Simm^20^, M. Capri^21^, C. Franceschi^21, 22^, PE. Slagboom^23^, DCS. Talbot^24^, C. Libert^25, 26^, S. Koskinen^27^, H. Bruunsgaard^28^, ÅM. Hansen^29, 30^, R. Lund^29,31^, M. Hurme^2,3^, M. Jylhä^1,2*^

^1^ Faculty of Social Sciences (Health Sciences), Tampere University, Tampere, Finland

^2^ Gerontology Research Center (GEREC), Finland

^3^ Faculty of Medicine and Health Technology (MET), Tampere University, Tampere, Finland

^4^ Department of Medical Epidemiology and Biostatistics, Karolinska Institutet, Stockholm, Sweden

^5^ Molecular Toxicology Group, University of Konstanz, Germany

^6^ BioTeSys GmbH, 73728 Esslingen, Germany

^7^ *deceased*; University of Namur, Research Unit on Cellular Biology, Rue de Bruxelles, 61, Namur B-5000, Belgium

^8^ Research Institute for Biomedical Aging Research, University of Innsbruck, Rennweg, 10, 6020 Innsbruck, Austria

^9^ Advanced Technology Center for Aging Research, Scientific Technological Area, IRCCS INRCA, Ancona, Italy

^10^ Nestlé Research, Nestlé Institute of Health Sciences, EPFL Innovation Park 1015 Lausanne, Switzerland

^11^ National Hellenic Research Foundation, Institute of Biology, Medicinal Chemistry and Biotechnology, Athens, Greece

^12^ Laboratory of the Molecular Bases of Ageing, Nencki Institute of Experimental Biology, Polish Academy of Sciences, 3 Pasteur street, 02-093 Warsaw, Poland

^13^ Carol Davila University of Medicine and Pharmacy, Bucharest, Romania

^14^ National Institute for Public Health and the Environment (RIVM), Centre for Health Protection, P.O. Box 1, 3720 BA Bilthoven, The Netherlands

^15^ Straticell, Science Park Crealys, Rue Jean Sonet 10, 5032 Les Isnes, Belgique

^16^ Institute of Nutritional Sciences (140), University of Hohenheim, 70593 Stuttgart, Germany

^17^ Department of Molecular Toxicology, German Institute of Human Nutrition Potsdam-Rehbruecke (DIfE), Nuthetal, Germany

^18^ Department of Nutritional Toxicology, Friedrich Schiller University Jena, Dornburger Str. 24, 07743 Jena

^19^ Institute of Nutritional Medicine (180), University of Hohenheim, 70593 Stuttgart, Germany

^20^ Department of Cardiothoracic Surgery, University Hospital Halle, Ernst-Grube Str. 40, D-06120 Halle (Saale), Germany

^21^ DIMES- Department of Experimental, Diagnostic and Specialty Medicine; CIG-Interdepartmental Center “L.Galvani”, ALMA MATER STUDIORUM, University of Bologna, 40126 Bologna, Italy

^22^ Department of Applied Mathematics of the Institute of ITMM, National Research Lobachevsky State University of Nizhny Novgorod, Russian Federation

^23^ Section of Molecular Epidemiology, Leiden University Medical Centre, Leiden, The Netherlands

^24^ Unilever Science and Technology, Beauty and Personal Care, Sharnbrook, United Kingdom

^25^ Center for Inflammation Research, VIB, Ghent, Belgium

^26^ Department of Biomedical Molecular Biology, Ghent University, Ghent, Belgium

^27^ National Institute for Health and Welfare, Finland

^28^ Department of Clinical Immunology, Rigshospitalet, University hospital of Copenhagen, Denmark

^29^ Department of Public Health, University of Copenhagen, Denmark

^30^ National Research Centre for the working environment, Copenhagen, Denmark

^31^ Center for Healthy Aging, University of Copenhagen, Denmark

**Supplementary information 2**

**Figures**


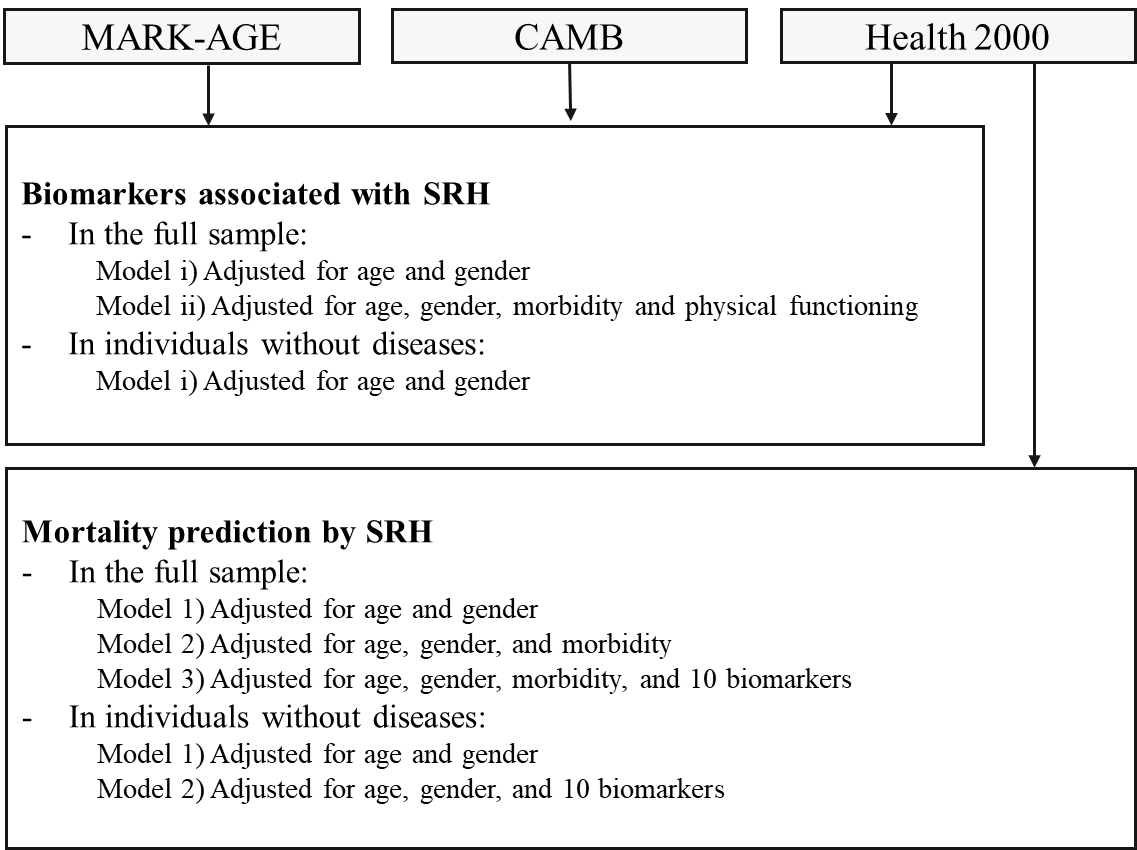


**Figure S1.** A schema of the analysis pipeline.

**
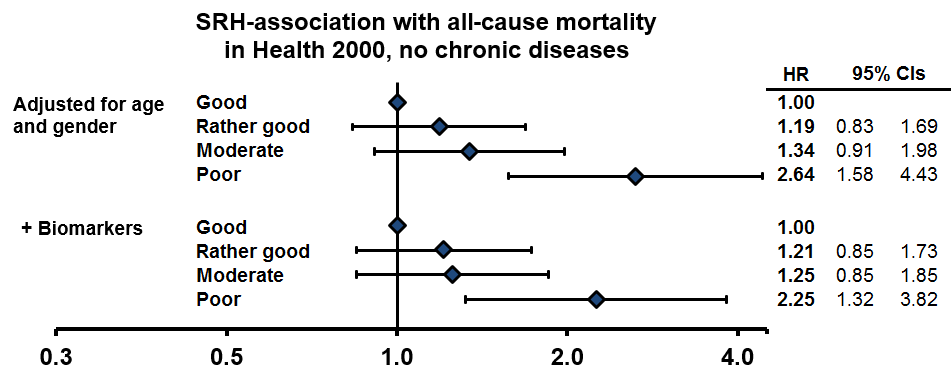
**

**Figure S2.**  The question whether the biomarkers significantly associated with SRH modify the association between SRH and mortality (**iii**) was explored also in the Health 2000 subsample without disease diagnoses (n=2,408). Of these individuals, 193 (8%) deceased during the follow-up of 15 years. Hazard ratios (HRs) and 95% CIs in the Cox proportional hazards models were adjusted for 1) age and gender, and 2) further for biomarkers (leptin, apolipoprotein B, cotinine, HbA1C, HDL:total cholesterol –ratio, HDL cholesterol, triglycerides, 25-hydroxy-vitaminD, gammaglutamyltransferase, and CRP). Good SRH was set as the reference category.

**Tables**

**Table S1. The numbers of biomarkers in the analysis and associated with SRH.** Associations between biomarkers and SRH in linear regression analysis, adjusted for i) age and gender and ii) additionally for number of diseases and physical functioning. A significant association was defined with the following criteria. 1) Association of biomarker that was available for analysis in at least two data sets: p<0.05 in all data sets, and 2) association of biomarker that was available for analysis in one data set: Bonferroni-adjusted p-value<0.05.

| **Number of biomarkers** | | | | |
| --- | --- | --- | --- | --- |
|  |  | **Associated with SRH** | | |
|  |  | **In all individuals** | | **In individuals**  **without diseases** |
|  | **In analysis** | **Model i (%)** | **Model ii (%)** | **Model i (%)** |
| Available in one data set | **133** | 46 (35) | 19 (14) | 8 (6) |
| Available in two data sets | **8** | 4 (50) | 2 (25) | 3 (38) |
| Available in three data sets | **9** | 7 (78) | 5 (56) | 5 (56) |
| **Total** | **150** | **57 (38)** | **26 (17)** | **16 (11)** |

**Table S2.** Numbers of biomarkers and their biological domains in the MARK-AGE, CAMB, and Health 2000 available for analysis and associated with SRH (adjusted for age and gender, model i).

|  | **In the analysis** | | | | | | **Associated with SRH (model i)** | | | | | |  |
| --- | --- | --- | --- | --- | --- | --- | --- | --- | --- | --- | --- | --- | --- |
| **Domain** | **MARK-AGE, CAMB,  Health 2000** | **MARK-AGE, Health 2000** | **Only in MARK-AGE** | **Only in CAMB** | **Only in  Health 2000** | **S**  **u**  **m** | **MARK-**  **AGE,**  **CAMB,  Health 2000** | **MARK-AGE,  Health 2000** | **Only in MARK-**  **AGE** | **Only in CAMB** | **Only in Health**  **2000** | **S**  **u**  **m** | |
| **lipid metabolism** | 5 | - | 21 | - | 2 | **28** | 4 | - | 15 | - | 2 | **21** | |
| **protein modification** | - | - | 19 | - | - | **19** | - | - | 3 | - | - | **3** | |
| **immune system** | 1 | - | 10 | 5 | 1 | **17** | 1 | - | 2 | - | 1 | **4** | |
| **nutrition** | - | 1 | 14 | - | - | **15** | - | 1 | 9 | - | - | **10** | |
| **oxidative stress** | - | - | 5 | - | - | **5** | - | - | 1 | - | - | **1** | |
| **lipid oxidation product; oxidative stress** | - | - | 4 | - | - | **4** | - | - | 1 | - | - | **1** | |
| **aminoacid**  **metabolism** | - | - | 3 | - | - | **3** | - | - | 1 | - | - | **1** | |
| **glucose metabolism** | 2 | 1 | - | - | - | **3** | 2 | 1 | - | - | - | **3** | |
| **selenium metabolism** | - | - | 3 | - | - | **3** | - | - | 3 | - | - | **3** | |
| **endocrine function** | - | - | - | - | 2 | **2** | - | - | - | - | 1 | **1** | |
| **one carbon cycle;  creatine metabolism** | - | - | 2 | - | - | **2** | - | - | 1 | - | - | **1** | |
| **purine metabolism** | - | - | 1 | - | 1 | **2** | - | - | - | - | 1 | **1** | |
| **transporter protein** | - | - | 1 | - | 1 | **2** | - | - | - | - | 1 | **1** | |
| **alcohol consumption;  liver function** | - | 1 | - | - | - | **1** | - | 1 | - | - | - | **1** | |
| **calcium status** | - | - | - | - | 1 | **1** | - | - | - | - | 1 | **1** | |
| **one-carbon metabolism;  CV health** | - | 1 | - | - | - | **1** | - | 1 | - | - | - | **1** | |
| **smoking exposure** | - | - | - | - | 1 | **1** | - | - | - | - | 1 | **1** | |
| **tissue damage** | - | - | 1 | - | - | **1** | - | - | 1 | - | - | **1** | |
| **wound healing,  tissue revascularization,  and repair** | - | - | 1 | - | - | **1** | - | - | 1 | - | - | **1** | |
| **iron metabolism** | - | 1 | 6 | - | - | **7** | - | 0 | 0 | - | - | **0** | |
| **byproduct of muscle  metabolism; renal health** | - | 2 | 1 | - | - | **3** | - | 0 | 0 | - | - | **0** | |
| **oxygen transfer** | 1 | - | 3 | - | - | **4** | 0 | - | 0 | - | - | **0** | |
| **copper metabolism** | - | - | 3 | - | - | **3** | - | - | 0 | - | - | **0** | |
| **metabolite** | - | - | 2 | - | - | **2** | - | - | 0 | - | - | **0** | |
| **protein metabolism** | - | - | 2 | - | - | **2** | - | - | 0 | - | - | **0** | |
| **protein modification;  advanced glycation  end product** | - | - | 2 | - | - | **2** | - | - | 0 | - | - | **0** | |
| **pyrimidine metabolism** | - | - | 2 | - | - | **2** | - | - | 0 | - | - | **0** | |
| **amino acid metabolism** | - | - | 1 | - | - | **1** | - | - | 0 | - | - | **0** | |
| **antiprotease** | - | - | 1 | - | - | **1** | - | - | 0 | - | - | **0** | |
| **copper and iron  metabolism** | - | - | 1 | - | - | **1** | - | - | 0 | - | - | **0** | |
| **copper and zinc  metabolism** | - | - | 1 | - | - | **1** | - | - | 0 | - | - | **0** | |
| **endocrine functions** | - | - | 1 | - | - | **1** | - | - | 0 | - | - | **0** | |
| **hormone,  neurotransmitter** | - | - | 1 | - | - | **1** | - | - | 0 | - | - | **0** | |
| **lipid metabolism;  lipid peroxidation  of polyunsaturated  fatty acids; oxidative stress** | - | - | 1 | - | - | **1** | - | - | 0 | - | - | **0** | |
| **lipid metabolism;  oxidative stress** | - | - | 1 | - | - | **1** | - | - | 0 | - | - | **0** | |
| **metabolism of  nitrogen-containing  compounds** | - | - | 1 | - | - | **1** | - | - | 0 | - | - | **0** | |
| **metabolite;**  **amine oxid** | - | - | 1 | - | - | **1** | - | - | 0 | - | - | **0** | |
| **zinc metabolism** | - | - | 1 | - | - | **1** | - | - | 0 | - | - | **0** | |
| **glucose and lipid  metabolism** | - | 1 | - | - | - | **1** | - | 0 | - | - | - | **0** | |
| **kalium intake status** | - | - | - | - | 1 | **1** | - | - | - | - | 0 | **0** | |
| **natrium and water  intake status** | - | - | - | - | 1 | **1** | - | - | - | - | 0 | **0** | |
| **Total** | **9** | **8** | **117** | **5** | **11** | **150** | **7** | **4** | **38** | **-** | **8** | **57** | |

**Table S3.** Distributions of participant characteristics in the three subsamples without diseases according to SRH.

|  |  | MARK-AGE |  | CAMB |  | Health 2000 |  |
| --- | --- | --- | --- | --- | --- | --- | --- |
|  | **Self-rated health** | *'good',  'very good' or 'excellent'* | *'poor'  or  'fair'* | *'good',  'very good' or 'excellent'* | *'poor'  or  'fair'* | *'moderate',  'rather good'  or 'good'* | *'poor'  or  'rather poor'* |
| Sample size | % (N) | 95 (1572) | 5.1 (85) | 97 (2418) | 3.2 (81) | 97 (2489) | 3.3 (85) |
| Age, years | Mean (Median) | 54 (54) | 56 (57) | 54 (56) | 53 (51) | 45 (43) | 52 (48) |
| Male | % (N) | 95 (773) | 4.7 (38) | 97 (1639) | 2.6 (43) | 96 (1147) | 4.4 (53) |
| Female | % (N) | 94 (799) | 5.6 (47) | 95 (779) | 4.7 (38) | 98 (1342) | 2.3 (32) |
| Number of diseases | N | - | - | - | - | - | - |
| Difficulties in physical  functioning,  sum of scores | Mean (Median) | 1.5 (1) | 2.5 (3) | 1.1 (1) | 2.4 (2) | 1.0 (1) | 2.2 (2) |

**Table S4.** Biomarkers available in two or three data sets associated with SRH, adjusted for age and gender (model i) in participants without diseases. A significant association was defined with the following criteria: p<0.05 in all data sets.

|  | **MARK-AGE** | | **CAMB** | | **Health 2000** | |
| --- | --- | --- | --- | --- | --- | --- |
| **Biomarker** | **β** | **p** | **β** | **p** | **β** | **p** |
| **25-HydroxyVitaminD** | -0.0042 | 4.3x10^-07^ | - | - | -0.0067 | 3.40x10^-11^ |
| **Creatinine** | -0.0059 | 0.00052 | - | - | -0.0057 | 0.0010 |
| **CRP** | 0.032 | 5.2x10^-06^ | 0.019 | 3.5x10^-08^ | 0.012 | 0.017 |
| **Glucose** | 0.066 | 0.0069 | 0.024 | 0.040 | 0.056 | 0.044 |
| **HDL cholesterol** | -0.23 | 2.6x10^-05^ | -0.24 | 1.7x10^-09^ | -0.16 | 0.00057 |
| **HDL:Total cholesterol, ratio** | -1.03 | 3.8x10^-04^ | -0.012 | 8.4x10^-09^ | -0.0085 | 0.00033 |
| **Insulin** | 0.027 | 5.5x10^-08^ | - | - | 0.0094 | 0.00026 |
| **Triglycerides** | 0.0015 | 7.1x10^-06^ | 0.063 | 1.5x10^-05^ | 0.078 | 1.5x10^-05^ |

Abbreviations: CRP=C-reactive protein; HDL = high-density lipoprotein.

**Table S5.** Biomarkers available in one data set associated with SRH, adjusted for age and gender (model i) in participants without diseases. A significant association was defined with the following criteria: Bonferroni-adjusted p-value<0.05.

| **Data set** | **Biomarker** | **β** | **p** |
| --- | --- | --- | --- |
| **MARK-AGE** | **Beta-carotene** | -0.23 | 3.2x10^-07^ |
| **MARK-AGE** | **Beta-cryptoxanthin** | -0.28 | 0.00030 |
| **MARK-AGE** | **Lutein** | -0.46 | 0.00014 |
| **MARK-AGE** | **Selenium** | -0.0070 | 7.9x10^-14^ |
| **MARK-AGE** | **Selenium bound to Albumin or Selenoprotein P** | -0.0077 | 3.6x10^-13^ |
| **MARK-AGE** | **Selenium bound to Glutathione Peroxidase** | -0.018 | 2.8x10^-06^ |
| **Health 2000** | **Cotinine** | 0.00034 | 2.5x10^-07^ |
| **Health 2000** | **Leptin** | 0.0058 | 3.0x10^-05^ |

Abbreviations: LDL = low-density lipoprotein.
